# Chiral Plasmonic Surface Temperature Switching by Several Tens of Kelvins in Titanium Nitride Nanostructures

**DOI:** 10.1021/acs.nanolett.5c05212

**Published:** 2025-12-22

**Authors:** Kenji Setoura, Tomoya Oshikiri, Mamoru Tamura, Ken Morita, Hideki Fujiwara, Satoshi Ishii, Yusuke Fujii, Yasutaka Matsuo, Takuya Iida, Kohei Imura

**Affiliations:** † Department of Electrical Materials and Engineering, Graduate School of Engineering, 12744 University of Hyogo, Himeji, Hyogo 671-2280, Japan; ‡ Institute of Multidisciplinary Research for Advanced Materials, Tohoku University, Sendai, Miyagi 980-8577, Japan; § Research Institute for Electronic Science, 12810 Hokkaido University, Sapporo, Hokkaido 001-0021, Japan; ∥ School of Science, Kwansei Gakuin University, 1 Gakuen Uegahara, Sanda, Hyogo 669-1330, Japan; ⊥ Research Institute for Light-induced Acceleration System (RILACS), 13311 Osaka Metropolitan University, 1-2 Gakuencho, Nakaku, Sakai, Osaka 599-8570, Japan; # Department of Chemistry and Biochemistry, School of Advanced Science and Engineering, 13148 Waseda University, 3-4-1, Okubo, Shinjuku, Tokyo 169-8555, Japan; ∇ Faculty of Engineering, Hokkai-Gakuen University, 1-1, Nishi 11, Minami 26, Chuo-ku, Sapporo 064-0926, Japan; ○ International Center for Materials Nanoarchitectonics (MANA), National Institute for Materials Science (NIMS), Tsukuba, Ibaraki 305-0044, Japan; ◆ Graduate School of Science and Technology, University of Tsukuba, Tsukuba, Ibaraki 305-8577, Japan; ¶ Graduate School of Chemical Sciences and Engineering, 12810 Hokkaido University, N13, W8, Kita-ku, Sapporo 060-8628, Japan; †† Department of Physics, Graduate School of Science, Osaka Metropolitan University, 1-2 Gakuencho, Nakaku, Sakai, Osaka 599-8570, Japan

**Keywords:** Thermoplasmonics, plasmonic heating, refractory plasmonics, chiral plasmonics, circular dichroism

## Abstract

The strong asymmetric optical response of plasmonic metal nanostructures to right- and left-handed circularly polarized light has attracted great interest in nanotechnology. However, when considering heat generation in these structures, the surface temperature distribution becomes nearly isothermal regardless of which handedness of circularly polarized light is used. This is because of the high thermal conductivity of noble metals and the diffusive nature of heat transfer. In this study, we experimentally show that the surface temperature patterns of chiral plasmonic nanostructures made from titanium nitride, which has a thermal conductivity less than 10% that of gold, become clearly different under right- and left-circularly polarized light, with the temperature contrast reaching several tens of kelvins. This temperature switching allows nanoscale spatial control of photothermal chemical reactions. Our findings suggest a significant potential for shaping nanoscale temperature distributions in the field of thermoplasmonics.

## Graphical abstract

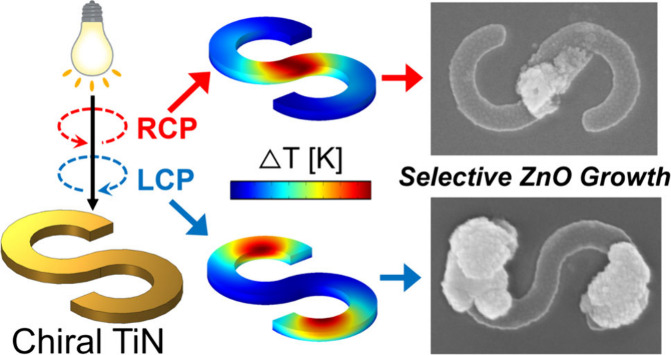

Metal nanostructures that exhibit localized surface plasmon resonance (LSPR) show two main functions when illuminated by light: the optical antenna effect^1^ and the role as nanoheaters.^2^ The latter has been widely studied in the field of thermoplasmonics, with reported applications to hyperthermia therapy,^3^ catalytic reactions,^4^ solar vapor generation,^5,6^ convective assembly,^7,8^ macroscopic photothermal reactions,^9–11^ and thermophoretic manipulation.^12–14^ A recent hot topic in this field is “chirality in plasmonic heating.” Examples include the circular dichroism in absorption and heat generation of Γ-shaped silver nanostructures,^15^ temperature measurements of chiral gold nanohelicoid particles under right- and left-handed circularly polarized light,^16,17^ and photothermal imaging of chiral nanostructures using the thermal lens effect of solvents.^18^ This trend corresponds to the growing interest in the chiral optical responses of metallic and dielectric nanostructures in nanophotonics and nanotechnology.^19–21^ However, chirality in thermoplasmonics has a fundamental limitation: the surface temperature patterns on nanostructures become nearly isothermal regardless of the handedness of the circular polarization. For example, in simulations of a Γ-shaped silver nanostructure with a length of 350 nm under right-handed circularly polarized illumination at an excitation wavelength of 860 nm, the maximum temperature rise (Δ*T*_max_(RCP)) was ≈16.9 K and the minimum (Δ*T*_min_(RCP)) was ≈15.7 K on the nanostructure surface. The ratio Δ*T*_min_/Δ*T*_max_ is 93%, indicating that the surface temperature distribution across the nanostructure is nearly uniform.^15^ A similar ratio was obtained when the nanostructure was illuminated with left-handed circularly polarized light under the same conditions. A very recent study reported the direct measurement of the surface temperature patterns of a single 200 nm gold nanohelicoid illuminated with circularly polarized light using atomic force microscopy (AFM) with a sensitivity of 0.1 K.^17^ The surface temperature imaging showed that when the overall temperature rise of the nanohelicoid was about 1 K, a nonuniform temperature distribution on the order of the detection sensitivity was observed. This slightly nonuniform surface temperature pattern was clearly found to switch depending on the handedness of the circularly polarized light. The surface temperature difference measured by AFM is estimated to be comparable to that of the Γ-shaped silver nanoparticles mentioned above. These results indicate that, due to the diffusive nature of heat transport, the surfaces of small structures tend to be nearly isothermal.^22^

In this study, to achieve large surface temperature differences through chiral plasmonic switching, we focused on titanium nitride (TiN), a material commonly used in refractory plasmonics.^23^ Titanium nitride shows LSPR in a wavelength range close to that of gold and has an exceptionally high bulk melting point of about 3200 K, making it an excellent optical material.^24^ On the other hand, an important point is that its thermal conductivity is 29 W m^–1^K^–1^,^25^ which is less than 10% of that of gold (314 W m^–1^K^–1^). In our previous numerical simulations, we found that using such a low-conductivity optical material allows the spatial characteristics of plasmon modes to be clearly imprinted on the temperature distribution, leading to surface temperature differences exceeding 100 K.^26,27^ In this Letter, based on this concept, we designed an S-shaped TiN nanostructure that shows chiral photothermal responses, fabricated it by electron-beam lithography, and performed laser irradiation experiments. We demonstrate that circularly polarized illumination can induce chiral plasmonic switching of highly nonuniform surface temperature patterns: the ratio of minimum to maximum surface temperatures reaches 56%, and the absolute temperature difference is remarkably large, on the order of several tens of kelvins.

Here, we first designed the photothermal response of TiN S-shaped nanostructures by the finite element method (FEM), and then present experimental results of circularly polarized laser irradiation on the fabricated nanostructures. Figure 1a shows the geometry of the system used in the FEM calculations. The S-shaped TiN nanostructure was placed on a sapphire substrate, with water as the superstrate. The sapphire substrate acts as a heat sink for the S-shaped nanostructures because of its high thermal conductivity. This role as a heat sink is later evaluated quantitatively by comparing it with a more commonly used glass substrate. The fabrication process of the S-shaped structure will be described later, but its dimensions are as follows: as shown in the scanning electron microscope (SEM) image in Figure 1b, the overall length is about 770 nm, the line width is 100 nm, and the thickness is 40 nm. We consider the case where the S-shaped nanostructure is illuminated from the top (−*z* direction) by a monochromatic circularly polarized plane wave. The calculation procedure is briefly described here, while details are given in our previous works.^26,27^ The governing equations are the frequency-domain Maxwell equations for nonmagnetic materials and the steady-state heat conduction equation. First, the optical response of the nanostructure to circularly polarized plane waves was obtained by solving Maxwell’s equations, giving the spatial distribution of the electric field. Once the plasmonic polarization pattern is determined, polarization currents induce the Joule heating effect, which generates localized heat inside the nanostructure.^28^ By solving steady-state heat conduction with this spatially inhomogeneous Joule heat as the source, the spatial pattern of the “chiral plasmon modes” is imprinted on the surface temperature of the S-shaped nanostructure. As boundary conditions, the outer region for Maxwell’s equations was surrounded by perfectly matched layers, while the outer boundary for steady-state heat conduction was set at room temperature (293 K), as shown in Figure 1a. The constant refractive indices of water and sapphire substrate were set to 1.33 and 1.77, respectively. The dielectric function of TiN was taken from the measured values of the thin film we deposited by sputtering (Supporting Information S1). The thermal conductivities of water, sapphire, and TiN were set to 0.6, 42, and 29 W m^–1^ K^–1^, respectively. Since the experiments employed CW laser irradiation on the S-shaped nanostructures, the calculations also treated steady-state solutions.

**Figure 1:**
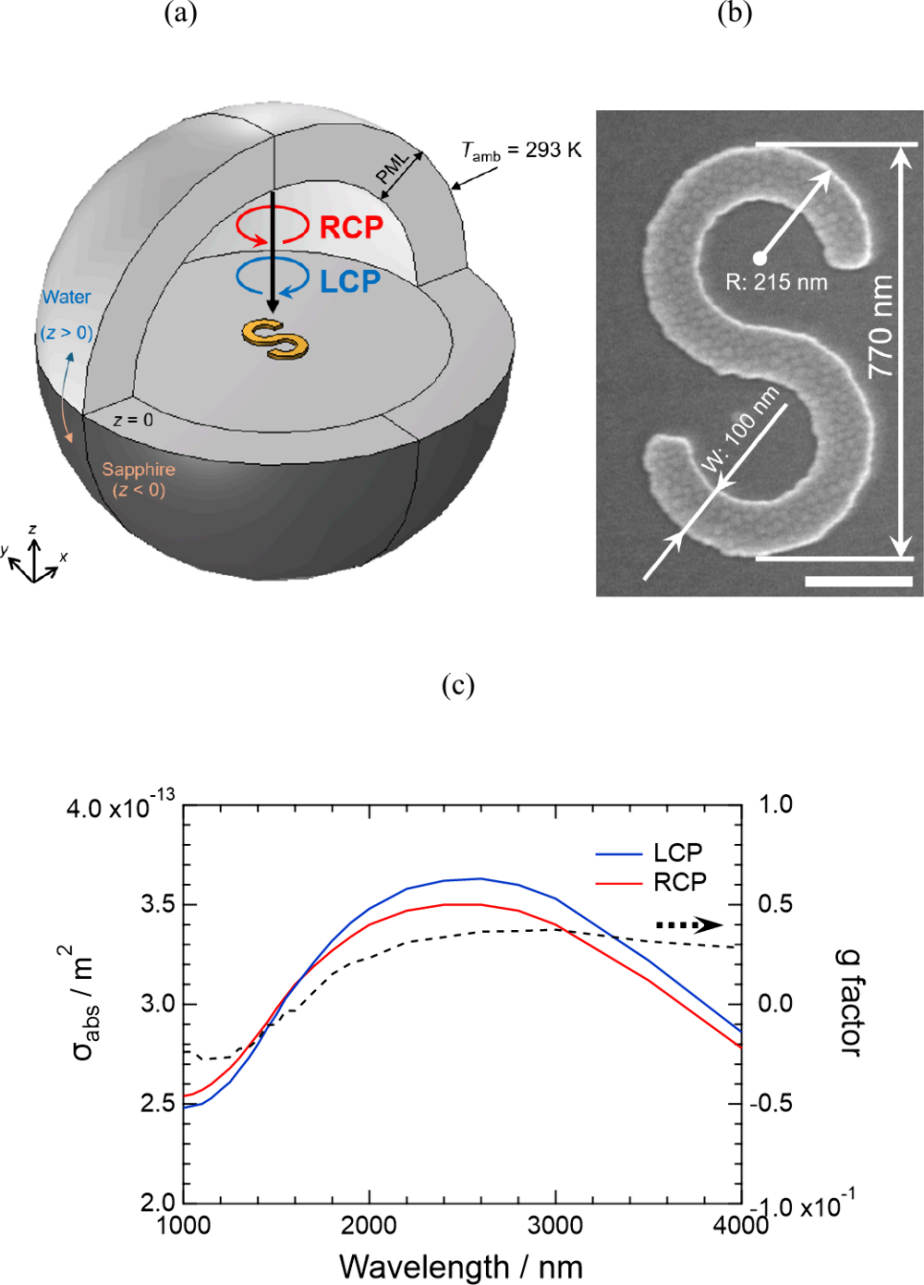
(a) Geometry of the simulation, where a TiN S-shaped nanostructure with a total length of 770 nm is illuminated by circularly polarized light. (b) SEM image showing the dimensions of the S-shaped nanostructure. Scale bar: 200 nm. (c) Calculated absorption cross-section spectra of the S-shaped nanostructure under RCP and LCP illumination. The g factor is shown by the dashed line on the right vertical axis.

Figure 1c shows the calculated absorption cross-section spectra when the S-shaped nanostructure is illuminated with right- and left-handed circularly polarized (RCP and LCP) light at various wavelengths. The rotation directions of RCP and LCP are shown in Figure 1a. The structure exhibits slightly different absorption for the two circular polarizations, reflecting its chiral plasmonic response. Because the overall length of the nanostructure is relatively large (770 nm), a broad resonance band appears around wavelengths of 1200–4000 nm. From these absorption spectra, we calculated the g-factor using the expression (σ_LCP_ − σ_RCP_)/((σ_LCP_ + σ_RCP_)/2), and the results are shown as a dashed line in Figure 1c. The g-factor of this S-shaped nanostructure reaches at most 0.03. In comparison, Γ-shaped silver nanostructures of similar size with a back reflector, designed for chiral photothermal conversion, exhibit g-factors ranging from 0.1 to 0.4,^15^ indicating that the optical anisotropy of the present S-shaped structure is relatively small. Nevertheless, due to the low thermal conductivity of TiN, the surface temperature of the nanostructure changes dramatically under circularly polarized light, as will be shown in detail later. From this broad absorption band, we focused on the wavelength of 1550 nm and carried out more detailed calculations of chiral temperature switching of plasmons. There are two reasons for using the wavelength of 1550 nm: (i) the absorption cross sections for RCP and LCP are almost identical, allowing rigorous verification of surface temperature switching under the same laser power, and (ii) we already have a continuous-wave laser at this wavelength for optothermal manipulation, which made the experiments easier to perform.

Figure 2 shows the electric field normalized by the incident field ***E***_0_, surface charge, Joule heat from polarization currents, and steady-state temperature distribution of the S-shaped nanostructure and its surroundings, obtained from the previously described calculations of the electric field and steady-state heat conduction. The upper panels correspond to RCP, and the lower panels to LCP. All the mappings in Figure 2 represent cross sections at the TiN–water interface (*z* = 40 nm) in the *x*–*y* plane, viewed from the water side. The light intensity was 1.0 × 10^10^ W m^–2^. From the electric fields in Figure 2a and e, it is seen that under RCP the field enhancement is approximately 2-fold along the edges of the S-shaped nanostructure, whereas under LCP the enhancement is localized at both ends of the S-shape and reaches about 5. The corresponding surface charge densities were calculated from Gauss’s law^27^ and are shown in Figure 2b and f. These results indicate that different plasmon modes are excited depending on the handedness of the circular polarization. A more detailed assignment of the plasmon modes was carried out using other finite element solvers, CST Studio Suite (https://www.3ds.com/products/simulia/cst-studio-suite) and FreeFEM++, for mode analysis and eigenfunction calculations, as described in .^29,30^

**Figure 2:**
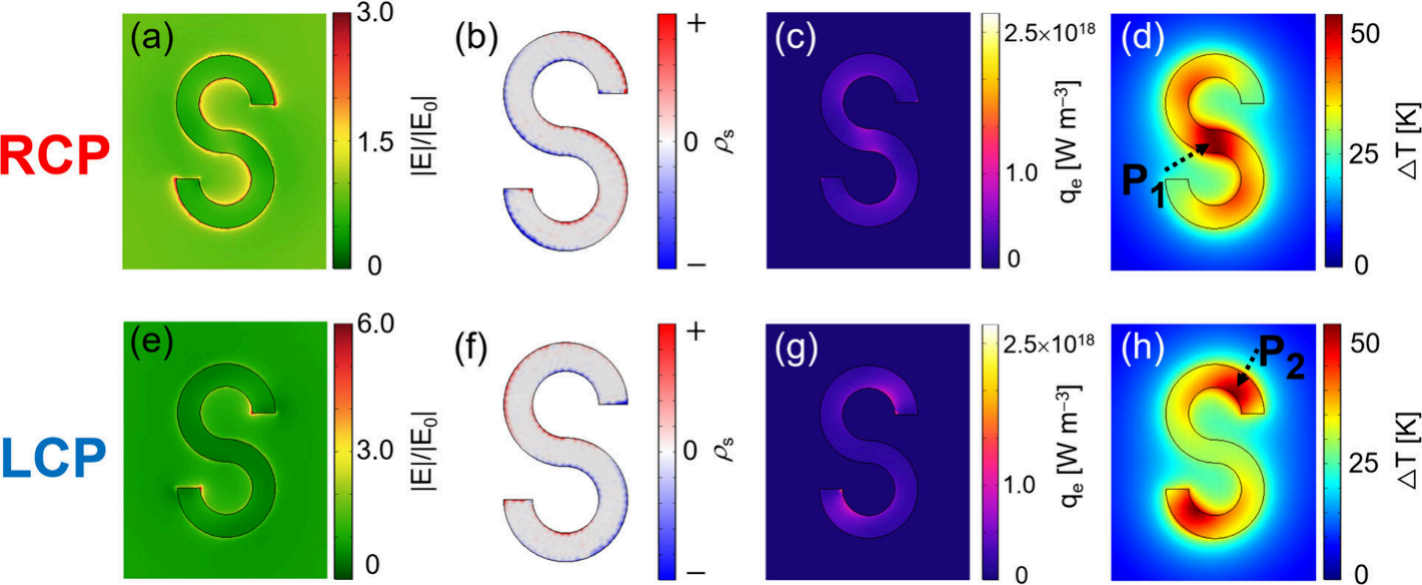
(a, e) Electric field distributions normalized by the incident field ***E***_0_. (b, f) Surface charge density distributions calculated using Gauss’s law. (c, g) Joule heat generated from polarization currents inside the nanostructure. (d, h) Steady-state temperature distributions on and around the surface of the S-shaped nanostructure. All figures represent *x*–*y* cross sections at *z* = 40 nm from the sapphire surface. The upper panels correspond to RCP illumination, and the lower panels correspond to LCP illumination. The light intensity was set to 1.0 × 10^10^ W m^–2^, and the excitation wavelength was 1550 nm.

As reported in earlier simulations of plasmonic heating, the location of local heat generation strongly depends on the plasmon mode.^28^ In Figure 2c (RCP), heat is generated mainly at the center of the S-shape, while in Figure 2g (LCP), it appears at both ends of the S-arms. Since the steady-state temperature distribution essentially follows the mapping of the heat sources, the same trend is observed in Figure 2d and h: under RCP only the center of the S-shape is selectively heated, while under LCP only the ends of the arms show a temperature rise. Remarkably, the surface temperature of the S-shaped nanostructure is completely switched between RCP and LCP, and in each case the maximum temperature difference on the nanostructure surface reaches as much as 30 K. Here, we quantitatively evaluate the surface temperature differences of the nanostructures. Under RCP illumination, the maximum temperature rise occurred at point P1 (0, 0, 40 nm) in Figure 2d, while the minimum temperature rise appeared at both ends of the S-shaped arms. Please note that the origin of the *x*–*y* coordinates corresponds to point P1 at the center of the S-shaped nanostructure (Figure 2d), and that *z* = 0 refers to the water–sapphire interface, as already shown in the calculation geometry in Figure 1a. Under LCP illumination, the maximum temperature rise occurred at point P2 (90 nm, 260 nm, 40 nm) in Figure 2h, and the temperature rise at P1 was the smallest. Therefore, we calculated the surface temperature ratio by dividing the temperature rise at P2 by that at P1 for RCP, and by dividing the temperature rise at P1 by that at P2 for LCP. The resulting ratios were Δ*T*_P2_/Δ*T*_P1_ = 63% for RCP and Δ*T*_P1_/Δ*T*_P2_ = 56% for LCP. These surface temperature ratios remain constant regardless of the incident light intensity. In water, individual nanostructures under steady-state CW laser heating do not induce bubble formation until about 220 °C,^31^ so with higher light intensity the surface temperature difference in TiN S-shaped nanostructures could reach 100 K or higher, based on the above estimation of the surface temperature ratios. Such a large temperature difference cannot be achieved in noble metal nanostructures with high thermal conductivity.^22,32^ Up to this point, we have mainly discussed the calculation results at 1550 nm. For comparison, the steady-state temperature distributions at a longer wavelength of 2400 nm and a shorter wavelength of 800 nm are shown in Supporting Information S3. Briefly, excitation at 2400 nm provided a clearer contrast in chiral temperature switching than at 1550 nm. In contrast, when the structure was illuminated at 800 nm, higher-order plasmon modes were excited, causing the heat generation sites to split into multiple locations and leading to a nearly uniform surface temperature of the S-shaped nanostructure.

We next performed heat-conduction calculations in which the thermal conductivity of the S-shaped nanostructure (*k*_metal_) and that of the substrate (*k*_sub_) were treated as variables. The purpose was to quantitatively examine the advantage of TiN over Au in shaping the surface temperature distribution, as well as the role of the substrate as a heat sink. In the following calculations, the dielectric function of the S-shaped nanostructure was fixed to that of TiN shown in Supporting Information S1, and the excitation wavelength was set to 1550 nm. Using the dielectric function of Au or changing the excitation wavelength would be possible options, but such changes would significantly alter the electric-field distribution and plasmon polarization patterns shown in Figure 2. Therefore, we kept the plasmonic mode fixed at 1550 nm circularly polarized excitation (as in Figure 2), and varied only *k*_metal_ and *k*_sub_ in the steady-state heat-conduction simulations. The refractive index of the substrate was also fixed at that of sapphire, *n* = 1.77, and the superstrate remained water. For the *k*_metal_, we used 314 W m^–1^ K^–1^ for Au and 29 W m^–1^ K^–1^ for TiN. For the *k*_sub_, we used 1.0 W m^–1^ K^–1^ for glass and 42 W m^–1^ K^–1^ for sapphire. The resulting steady-state temperature distributions are shown in Figure 3. These two-dimensional temperature maps correspond to a cross-section at *z* = 40 nm. The incident intensity was adjusted so that the maximum temperature rise on the nanostructure surface became approximately 53 K (the exact value is noted in the figure caption).

**Figure 3:**
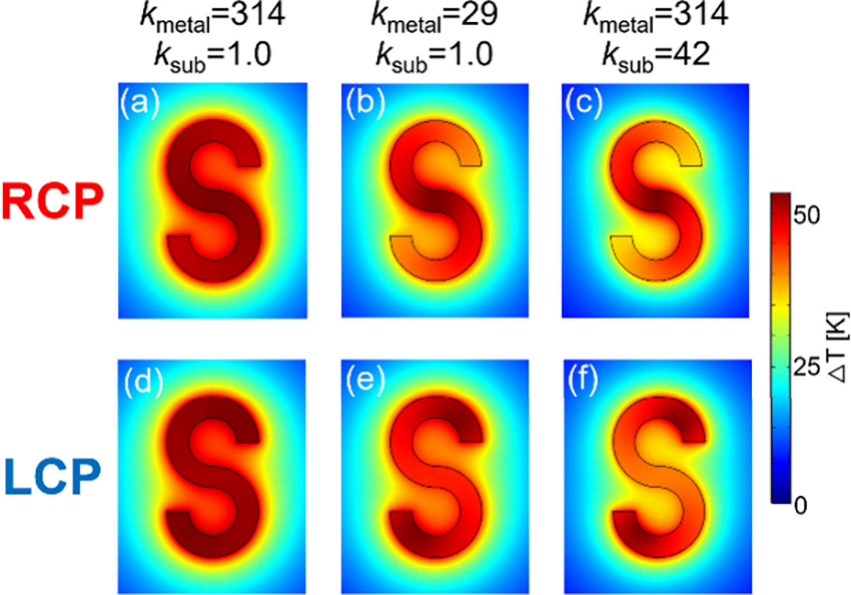
Calculated steady-state temperature distributions on and around the surface of the S-shaped nanostructure. All figures represent *x*–*y* cross sections at *z* = 40 nm from the substrate surface. The upper panels correspond to RCP illumination, and the lower panels correspond to LCP illumination. The excitation wavelength was 1550 nm. (a, d) The thermal conductivities of *k*_metal_ = 314 W m^–1^ K^–1^ and *k*_sub_ = 1.0 W m^–1^ K^–1^ were used, and the light intensity was set to 7.4 × 10^8^ W m^–2^. (b, e) The thermal conductivities of *k*_metal_ = 29 W m^–1^ K^–1^ and *k*_sub_ = 1.0 W m^–1^ K^–1^ were used, and the light intensity was set to 7.4 × 10^8^ W m^–2^. (c, f) The thermal conductivities of *k*_metal_ = 314 W m^–1^ K^–1^ and *k*_sub_ = 42 W m^–1^ K^–1^ were used, and the light intensity was set to 1.5 × 10^10^ W m^–2^.

We first focus on Figure 3a and d, where the thermal conductivities of the nanostructure and substrate were Au and glass, respectively. Under both RCP and LCP illumination, the surface of the nanostructure became nearly isothermal. In contrast, when a TiN nanostructure was placed on a glass substrate (Figure 3b and e), the surface temperature became clearly nonuniform for both RCP and LCP illumination. Most notably, even when the nanostructure was made of highly conductive Au, a clear temperature contrast was obtained if it was placed on a sapphire substrate (Figure 3c and f). These three combinations of thermal conductivities demonstrate that, for thin two-dimensional nanostructures on substrates, the heat-sink function of a high-thermal-conductivity substrate plays a significant role. To quantitatively evaluate the surface temperature patterns, we computed the surface temperature ratios Δ*T*_P2_/Δ*T*_P1_ for RCP illumination and Δ*T*_P1_/Δ*T*_P2_ for LCP illumination using the same procedure as before, and summarized the values in Table 1. For comparison, the values for a TiN nanostructure on a sapphire substrate (Figure 2) are also included.

**Table 1:** Surface Temperature Ratios

	Δ*T*_P2_/Δ*T*_P1_ under RCP (%)	Δ*T*_P1_/Δ*T*_P2_ under LCP (%)
*k*_metal_ = 314: *k*_sub_ = 1.0	95	97
*k*_metal_ = 29: *k*_sub_ = 1.0	79	86
*k*_metal_ = 314: *k*_sub_ = 42	73	77
*k*_metal_ = 29: *k*_sub_ = 42	63	56

We now discuss the values in Table 1. For Au nanostructures on a glass substrate, the surface temperature ratios were 95–97% for both RCP and LCP illumination, close to values reported for Ag nanostructures.^15^ For TiN nanostructures on glass, the ratio was roughly 80%, which is low enough for the heat-conduction simulations to show a clearly nonuniform surface temperature pattern. On the other hand, even with a highly conductive Au nanostructure, placing a thin two-dimensional structure on a good heat sink yielded a distinctly nonuniform temperature distribution. While sapphire is widely used as a transparent substrate with high thermal conductivity, silicon carbide (SiC) is another option; it is a wide-bandgap semiconductor with a thermal conductivity of about 280 W m^–1^ K^–1^ and exhibits transparency in the visible range depending on the dopant species. Previous studies have reported that SiC can serve as an effective heat sink for optical nanoheating.^33^ The most remarkable values in Table 1 are the 63% and 56% obtained when TiN nanostructures were placed on a sapphire substrate. These ratios indicate that, for example, when the maximum surface temperature of the nanostructure is 100 °C, the colder region is about 60 °C. Such a large temperature difference is expected to enable spatial control of thermal reactions.

To perform experiments under conditions comparable to the calculations in Figure 2, S-shaped TiN nanostructures were fabricated by electron-beam lithography. The procedure followed literatures.^34,35^ Briefly, a 40 nm TiN film was deposited on a sapphire substrate (SS-2SC-2525, 25.4 mm × 25.4 mm × 0.5 mm, C plane, double sides polished, ALLIANCE Biosystems) by RF sputtering using a Ti target under Ar and N_2_ flow at 600 °C (JEC-SP360M, Jeol). The dielectric function of this TiN film was measured by a spectroscopic ellipsometer (FE-5000, Otsuka Electronics Co., Ltd., see Supporting Information S1), and used in the FEM calculations described above. On top of the TiN film, an electron-beam resist (ZEP520A) was spin-coated, and the S-shaped patterns were patterned by an electron beam lithography system (ELS–F125-U, ELIONIX). After developing the resist, a 50 nm Cr layer was deposited as a hard mask. The resist was lifted off, and the TiN film was dry-etched using Ar and Cl gas (RIE-101iPH, Samco),^36^ followed by wet etching to remove the Cr. This process yielded the TiN nanostructures shown in Figure 1b. All electron microscopy images were taken using a JSM-7610F (Jeol) after sputtering Pt onto the substrate with a fine coater (JEC-3000FC, Jeol). The S-shaped nanostructures were arranged in a square lattice with a pitch of 9 μm on the sapphire substrate. The optical setup used for the laser irradiation experiments is briefly described as follows. Figure 4a shows an inverted dark-field optical microscope (IX-73, Olympus), and an image of the array of S-shaped nanostructures observed with this system is presented in Figure 4b. In this setup, a CW laser at 1550 nm (FLH-1550–35-PM-B, Civil Laser) was converted to RCP or LCP using a quarter-wave plate (CP1R and CP1L, Thorlabs), and then focused onto individual S-shaped nanostructures by an objective lens (LCPLN50XIR, NA = 0.65, Olympus). The sapphire substrate, which can exhibit birefringence depending on the incident laser conditions, was placed on the upper side of the sample chamber as described later, shown in Figure 4a. The diameter of the focused laser spot was measured with a near-infrared camera (Cat# 56–567, Edmund Optics) to be 2.8 μm (as full width half-maximum: fwhm), which is sufficiently larger than the size of the S-shaped nanostructures.

**Figure 4:**
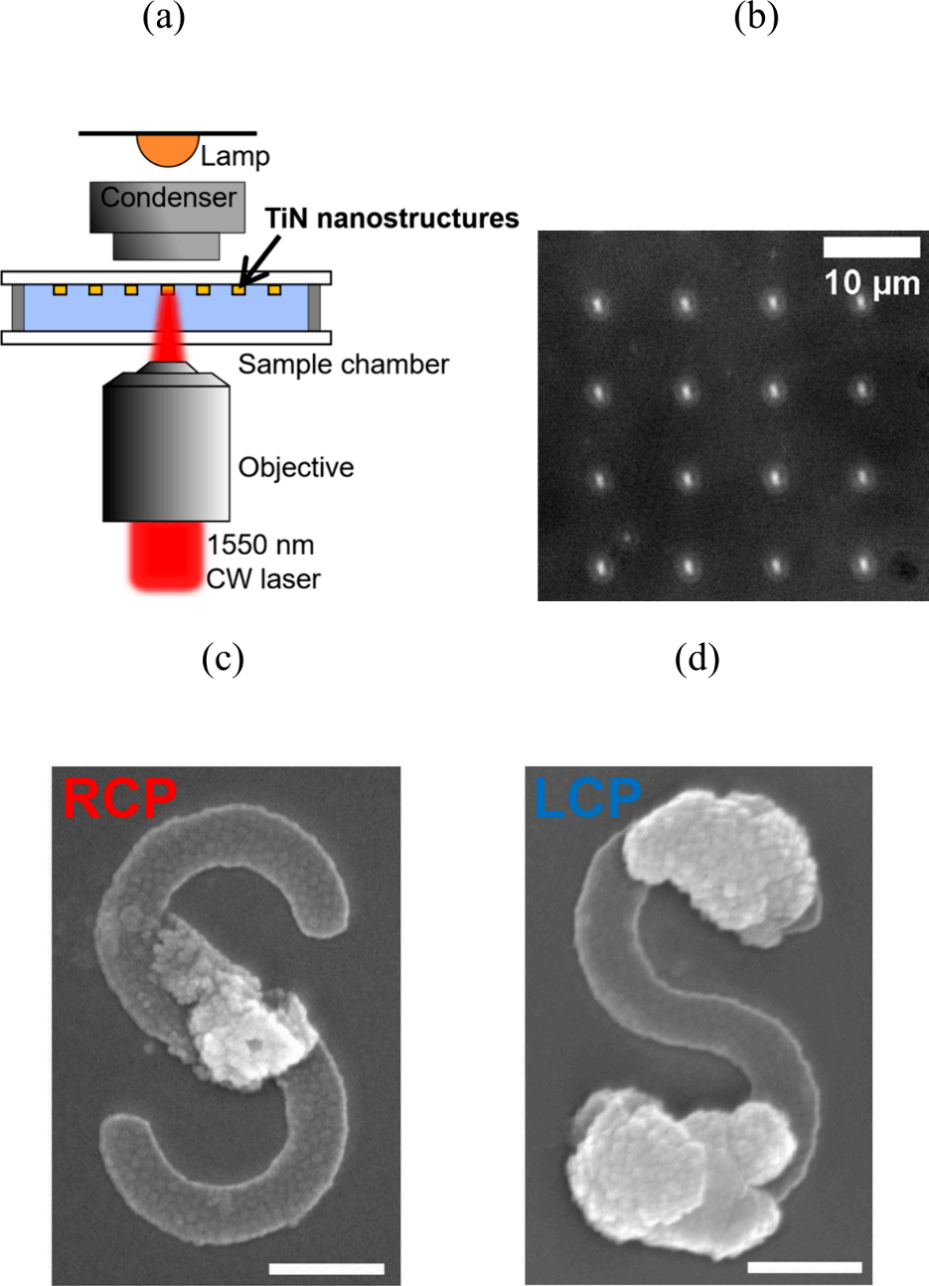
(a) Schematic of laser irradiation on an individual S-shaped nanostructure under dark-field optical microscopy. (b) Dark-field image of individual S-shaped nanostructures arranged with a 9 μm pitch. (c, d) SEM images of S-shaped nanostructures irradiated for 3 s with RCP or LCP laser light at a wavelength of 1550 nm. In both cases, the irradiance was 1.0 × 10^10^ W m^–2^. Scale bar: 200 nm.

The nanoscale temperature distributions shown in Figure 2d and h are difficult to image directly with an optical microscope because of the diffraction limit. To address this, we attempted to visualize these unique distributions by inducing a thermal reaction through optical heating of the nanostructures and then observing the products with SEM. A suitable process for this purpose is the hydrothermal synthesis of ZnO. It is known that bulk gold films and individual gold nanostructures can locally heat and trigger ZnO deposition from precursors in aqueous solution.^37,38^ In this study, we prepared precursor solutions by using deionized water as the solvent, mixing equal volumes of 75 mM zinc nitrate hexahydrate aqueous solution and 75 mM hexamethylenetetramine aqueous solution. A 40 μL droplet of this precursor solution was placed on a sapphire substrate. Using a 200 μm-thick silicone rubber spacer, the droplet was sandwiched with a borosilicate glass coverslip (C024321, Matsunami Glass Ind. LTD) to form the sample chamber shown in Figure 4a. Circularly polarized laser irradiation was applied to individual S-shaped nanostructures for 3 s. The irradiance in the laser spot was 1.0 × 10^10^ W m^–2^, which is the same as the incident light intensity used in Figure 2. During irradiation, dark-field observation showed an increase in light scattering associated with ZnO formation (see Supporting Information Video S1).

SEM images of the S-shaped nanostructures after irradiation with RCP and LCP are shown in Figure 4c and d. With RCP, the thermal products were selectively deposited at the center of the S-shaped structure, whereas with LCP, they appeared at both ends of the arms. This striking result contrasts sharply with the case of gold nanostructures, where ZnO tends to coat the entire structure.^38^ To demonstrate the reproducibility of ZnO deposition under identical laser irradiation conditions, we included more than ten SEM images each for RCP and LCP in Supporting Information S4. Since several SEM images for both RCP and LCP showed good agreement with the calculated nonuniform temperature patterns, we consider the reproducibility of this experiment to be sufficient. Under these irradiation conditions, the maximum Δ*T* values in Figure 2d and h were about 50 K, which is consistent with the literature on ZnO hydrothermal synthesis induced by gold nanostructure heating,^38^ and thus reasonable. Elemental mapping was performed using the energy-dispersive X-ray spectroscopy (EDS) during SEM observation. The resulting 2D Zn map confirmed that the Zn signal was localized to the product on the S-shaped nanostructures, suggesting the deposits are ZnO (Supporting Information S5).

Before presenting the conclusion, several remarks should be noted in this Letter. First, because water exhibits moderate absorption at a wavelength of 1550 nm,^39^ the background heating of water cannot be ignored. However, under the irradiation conditions shown in Figure 4, we applied the laser to the precursor solution without nanostructures, and no ZnO formation was observed by either optical microscopy or SEM. On the other hand, weak natural convection driven by water absorption was observed under the optical microscope. If the background water temperature were sufficiently high, one might be concerned that ZnO microcrystals formed in the bulk solution could be transported by convection and deposited onto the TiN nanostructures. However, as noted above, ZnO formation does not occur in the background solution at this laser intensity. Therefore, we consider the contribution of natural convection to be minor in this study.

Finally, we discuss the size of the S-shaped nanostructures used in this Letter. Simulations similar to those in Figure 2 showed that chiral temperature switching also occurs in TiN S-shaped nanostructures with a total length of 400 nm and a line width of 50 nm. However, given our limited access to electron-beam lithography, fabricating such small structures with sufficient resolution was challenging. In contrast, when the total length exceeds 1 μm, the contribution of localized plasmons to light absorption decreases, making it difficult to achieve a clear temperature contrast that depends on the wavelength or polarization. Supplementary calculations regarding these size effects are provided in Supporting Information S6.

## Conclusions

In summary, we experimentally demonstrated nanoscale spatial control of photothermal chemical reactions on TiN S-shaped nanostructures that exhibit chiral temperature switching under circularly polarized light. The design of these nanostructures was first optimized using finite element method simulations, which revealed completely different surface temperature distributions under right- and left-circularly polarized light. We then fabricated the designed S-shaped nanostructures by electron-beam lithography and related nanofabrication techniques. To visualize the predicted chiral temperature distributions of individual nanostructures, we conducted hydrothermal synthesis of ZnO under optical microscopy while irradiating them with either right- or left-circularly polarized lasers. SEM images of the reaction products confirmed that the S-shaped nanostructures exhibit the chiral temperature switching as predicted by the simulations. This large temperature switching of several tens of kelvins cannot be achieved with noble-metal-based thermoplasmonic systems. Therefore, the findings of this Letter provide a useful basis for achieving more precise spatiotemporal control of plasmonic optofluidics and thermoplasmonic reactions.

## Supplementary Material





## Notes

Dielectric function spectra of TiN (S1), detailed characterization of the plasmon modes at 1550 nm (S2), steady-state temperature distributions of the S-shaped nanostructure at excitation wavelengths of 800 and 2400 nm (S3), supplementary SEM images of ZnO hydrothermal synthesis under RCP and LCP (S4), 2D EDS mapping of the products of the hydrothermal synthesis (S5), and the effect of size on chiral plasmonic temperature switching (S6) (PDF)
Temporal increase of light scattering associated with ZnO formation on the S-shaped nanostructure (MP4)
