# Supplementary material for: Chiral Plasmonic Surface Temperature Switching by Several Tens of Kelvins in Titanium Nitride Nanostructures
Source: Nano Lett. 2025 Dec 22;26(1):351–7. doi: 10.1021/acs.nanolett.5c05212 (PMC12810465; doi:10.1021/acs.nanolett.5c05212)
Supplement: Supplementary file 1 [file nl5c05212_si_001.pdf]

Supporting Information

"Chiral Plasmonic Surface Temperature Switching by Several Tens of Kelvins in Titanium Nitride Nanostructures"

Kenji Setoura[, 1], Tomoya Oshikiri[2, 3], Mamoru Tamura[4, 5], Ken Morita[6], Hideki Fujiwara[7], Satoshi Ishii[8, 9], Yusuke Fujii[10], Yasutaka Matsuo[3], Takuya Iida[5, 11], and Kohei Imura[6]*

1. Department of Electrical Materials and Engineering, Graduate School of Engineering, University of Hyogo, Himeji, Hyogo, 671-2280, Japan. ***Email: setoura@eng.u-hyogo.ac.jp**
2. Institute of Multidisciplinary Research for Advanced Materials, Tohoku University, Sendai, Miyagi, 980-8577, Japan.
3. Research Institute for Electronic Science, Hokkaido University, Sapporo, Hokkaido, 001-0021, Japan.
4. School of Science, Kwansei Gakuin University, 1 Gakuen Uegahara, Sanda, Hyogo 669-1330, Japan.
5. Research Institute for Light-induced Acceleration System (RILACS), Osaka Metropolitan University, 1-2 Gakuencho, Nakaku, Sakai, Osaka 599-8570, Japan.
6. Department of Chemistry and Biochemistry, School of Advanced Science and Engineering, Waseda University, 3-4-1, Okubo, Shinjuku, Tokyo, 169-8555, Japan
7. Faculty of Engineering, Hokkai-Gakuen University, 1-1, Nishi 11, Minami 26, Chuo-ku, Sapporo 064-0926, Japan.
8. International Center for Materials Nanoarchitectonics (MANA), National Institute for Materials Science (NIMS), Tsukuba, Ibaraki 305-0044, Japan
9. Graduate School of Science and Technology, University of Tsukuba, Tsukuba, Ibaraki 305-8577, Japan
10. Graduate School of Chemical Sciences and Engineering, Hokkaido University, N13, W8, Kita-ku, Sapporo, 060-8628, Japan.
11. Department of Physics, Graduate School of Science, Osaka Metropolitan University, 1-2 Gakuencho, Nakaku, Sakai, Osaka 599-8570, Japan.

S1. Dielectric function of titanium nitride.

We deposited titanium nitride (TiN) films on sapphire substrates at 600 ˚C by RF sputtering using a Ti target with Ar and N_2 gas flow (JEC-SP360M, Jeol). The dielectric function of this film was measured by spectroscopic ellipsometry in the wavelength range of 250–800 nm (**Figure S1**). The dielectric constant at 1550 nm, the wavelength of interest in the present simulations, was obtained by fitting the measured dielectric response with the Drude–Lorentz model.

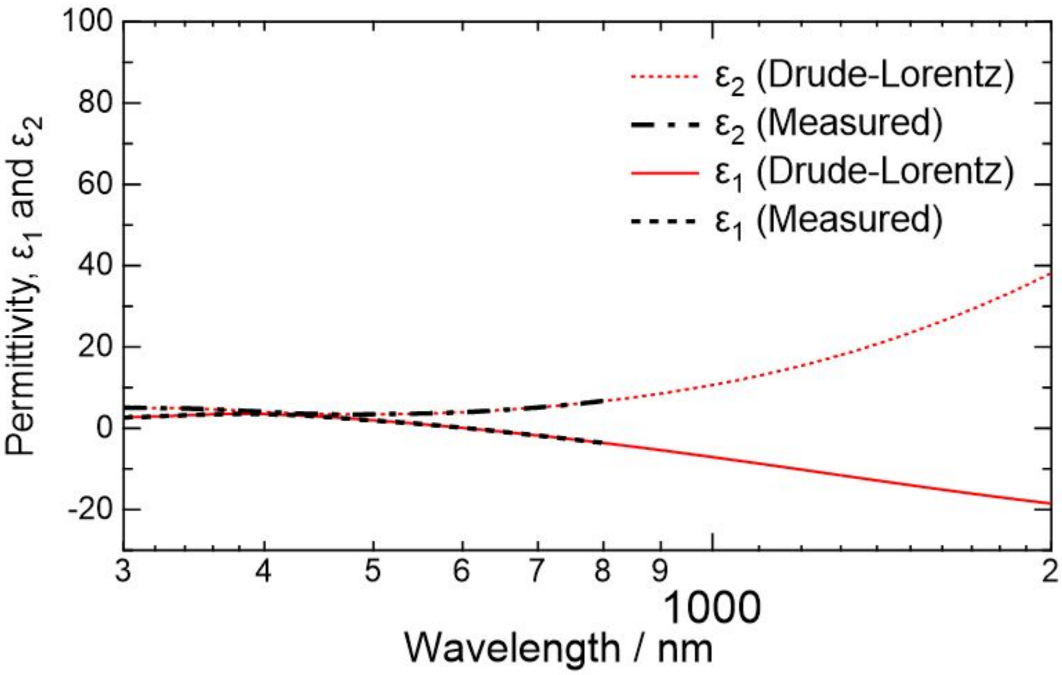

Figure S1. Dielectric function spectra measured by spectroscopic ellipsometry and fitted using the Drude–Lorentz model.

S2. Detailed characterization of the plasmon modes at 1550 nm.

We examine the optical properties of an S-shaped TiN nanostructure using electromagnetic field simulations based on the finite integration technique using CST studio suite [https://www.3ds.com/products/simulia/cst-studio-suite]. **Figure S2a** shows absorption cross-section spectra of the nanostructure for RCP and LCP incidence. The spectra show a peak near 2200 nm, in accordance with the one in **Figure 1c** of the main article. Absorption cross section for LCP incidence near the peak is larger than that for RCP. To reveal the physical origin of the difference, the surface current density on the structure was calculated, as shown in **Figure S2b and S2c**. We found that the surface current density is high at the center of the structure for RCP, while it is high at both ends of the nanostructure for LCP. Spatial distributions calculated in **Figure S2b and S2c** are nearly identical with temperature distribution observed in **Figure 2d and 2h** of the main article.

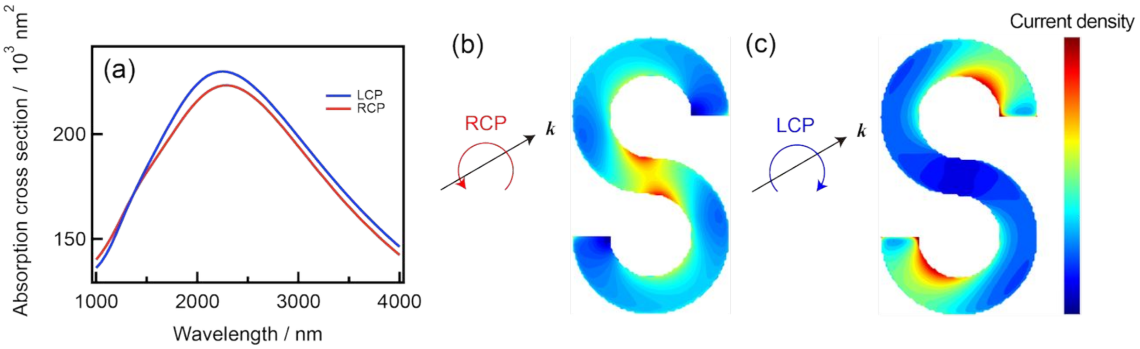

Figure S2 (a) Calculated far-field absorption spectra of the S-shaped TiN nanostructure for RCP (red) and LCP (blue) incidence. (b,c) Calculated surface charge density on the S-shaped nanostructure for RCP and LCP incidence, respectively.

We revealed from near-field imaging of plasmonic nanostructures that these unique spatial distributions are well correlated to the plasmon mode resonantly excited [Imaeda, K.; Hasegawa,

S.; Imura, K. *J. Phys. Chem. C* **2018**, 122, 7399–7409.]. To unravel the origin of the difference observed in **Figure S2b and S2c**, we calculated eigenfunctions for a particle confined in an S-shaped two-dimensional well using the finite element method [Hecht, F. *J. Numer. Math.* **2012**, 20, 251–265. https://doi.org/10.1515/jnum-2012-0013.]. **Figure S3** shows the five lowest eigenfunctions of the S-shaped nanostructure. Eigen energy is normalized with that of the lowest mode, E_0. We revealed from the calculation that the eigenenergies of these modes are very close to each other, indicating that these modes are spectrally overlapped in the absorption spectra. It is to be noted that the spatial distributions of **Figure S3c and S3d** are very similar to those in **Figure S2b and S2c**, respectively. These two modes are spectrally overlapped and thus are excited simultaneously at a given wavelength. The distinct spatial distributions calculated in **Figure S2b and S2c** indicate that these modes are selectively excited by RCP and LCP, respectively.

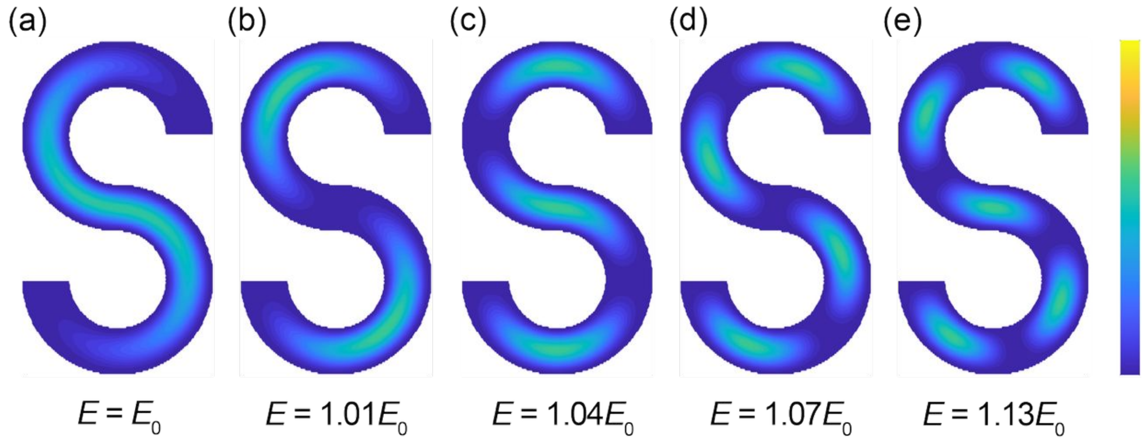

Figure S3 (a-e) Five lowest eigenfunctions calculated for a particle confined in an S-shaped two-dimensional potential well. The eigenenergy of each mode is normalized by that of the lowest one (E_0), and is shown below each mode.

S3. Steady-state temperature distributions of the S-shaped nanostructure at excitation wavelengths of 800, 1550, and 2400 nm.

Figure S4 shows the steady-state temperature distributions of the S-shaped nanostructure with a total length of 770 nm, excited at wavelengths of 800 and 2400 nm under the same irradiation conditions as those in **Figure 2** of the main article. For comparison, the result at 1550 nm from the main article is also shown. At 800 nm, higher-order plasmon modes are excited, generating multiple heat spots and resulting in a nearly uniform temperature distribution. In contrast, at 2400 nm, a clearer temperature contrast is obtained compared with the result at 1550 nm in the main article. The demonstration experiment was performed at 1550 nm due to the laser setup.

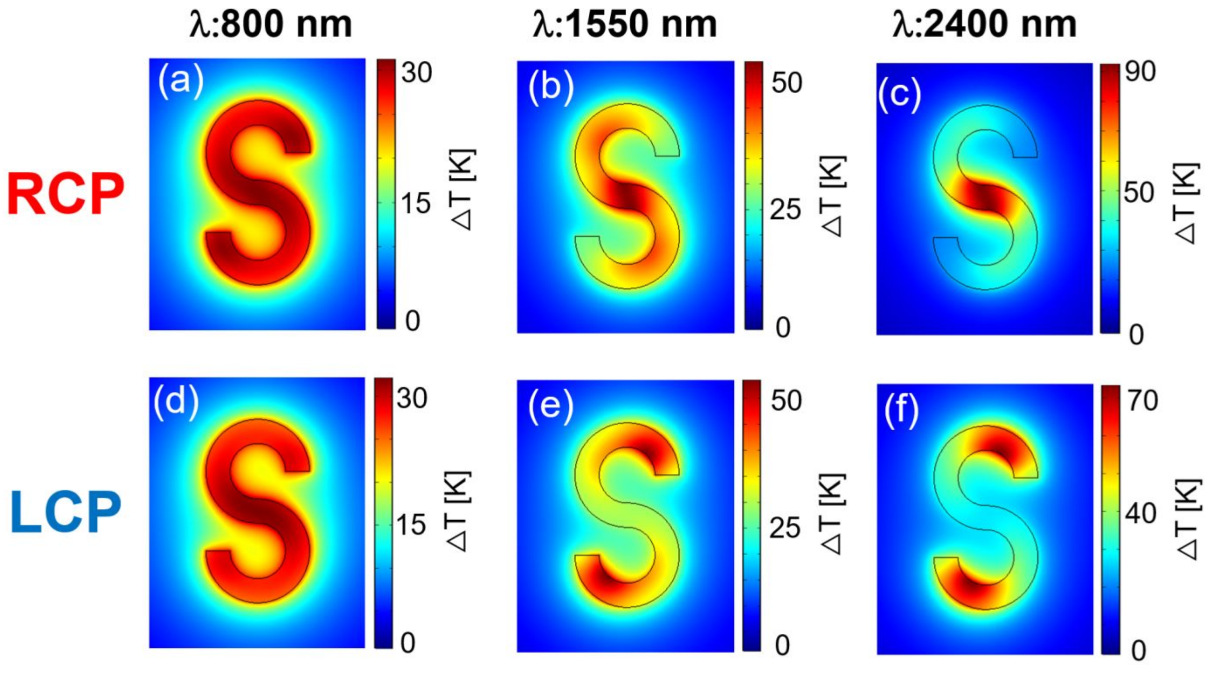

Figure S4. Steady-state temperature distributions of the S-shaped nanostructure with a total length of 770 nm under RCP and LCP illumination at each wavelength. All figures show *x*–*y* cross-sections at $z = 40$ nm. The irradiance was set to 1.0×10^{10} W m^{-2}, the same as in Figure 2 of the main article.

S4. Supplementary SEM images of ZnO hydrothermal synthesis under RCP and LCP.

Figure S5 shows SEM images confirming the reproducibility of ZnO nanohydrothermal synthesis under the same laser irradiation conditions as in **Figure 4** of the main article. The upper half, outlined in red, corresponds to RCP, and the lower half, outlined in blue, corresponds to LCP. For both RCP and LCP, ZnO deposition patterns that resembled the calculated surface temperature patterns in **Fig. 2d** and **2h** of the main article were judged as successful and placed in the upper panel, while those that did not were labeled as unsuccessful and placed in the lower panel. In each case, 8 out of 11 SEM images were successful. The unsuccessful results were either due to almost no ZnO formation or excessive ZnO growth during the 3-second laser exposure. When the amount of ZnO deposition was appropriate, the resulting ZnO pattern resembled the non-uniform temperature distribution, as shown in the SEM images of the successful cases. This reproducibility is expected to improve by optimizing experimental conditions, such as laser irradiation time, laser power, and the concentration of the ZnO precursor solution.

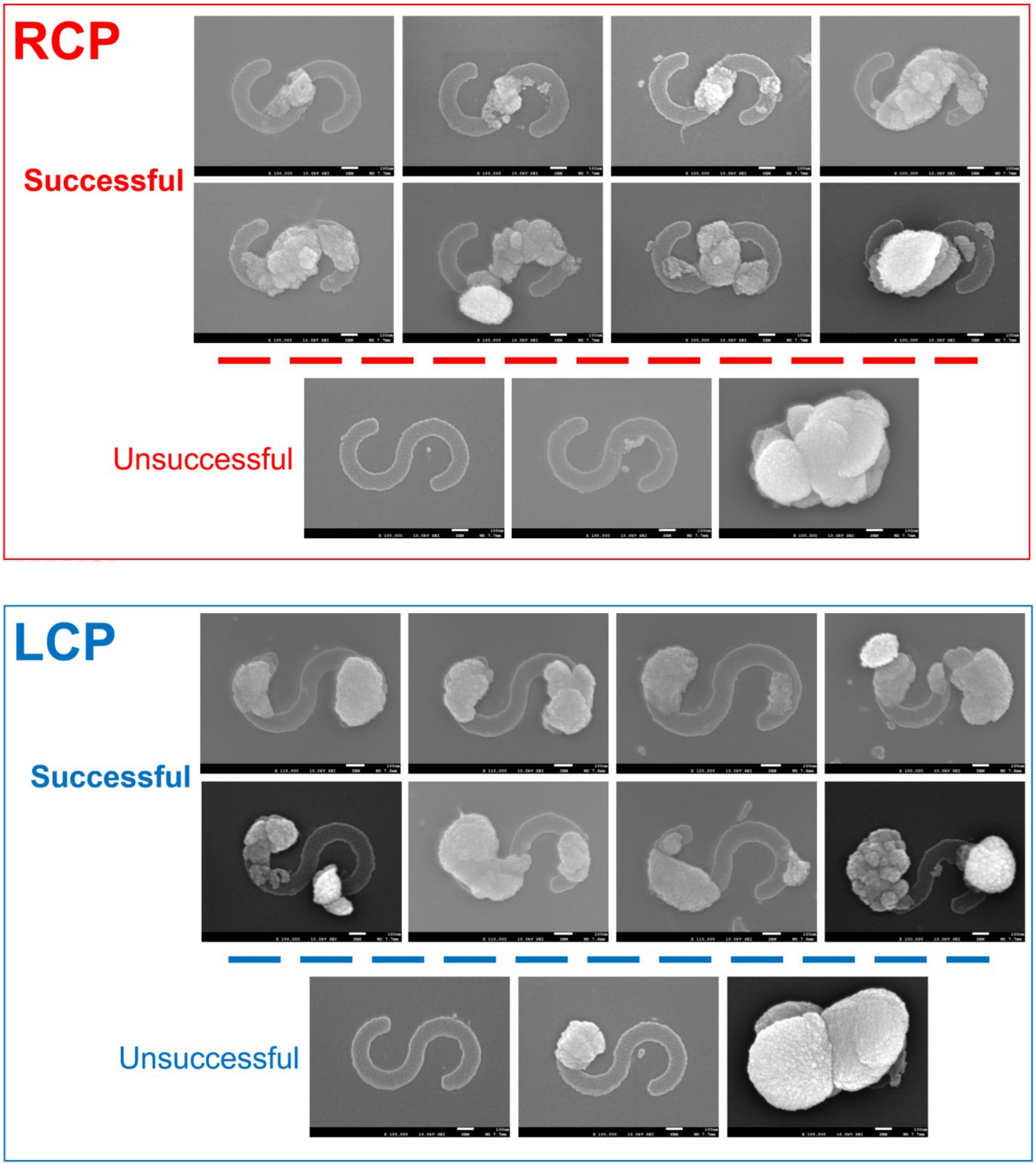

Figure S5. SEM images of ZnO hydrothermal synthesis on the S-shaped nanostructures under 1550 nm RCP and LCP laser illumination. The irradiation conditions are the same as in the main article. Scale bar: 100 nm. The total length of the S-shaped nanostructure is about 770 nm.

S5. Characterization of the hydrothermal synthesis products by EDS.

Two-dimensional (2D) elemental mapping of the products formed on the TiN S-shaped nanostructures under laser irradiation, as observed in the SEM images presented in the main article, was carried out using energy-dispersive X-ray spectroscopy (EDS). **Figure S6a** shows an SEM image of the S-shaped nanostructure irradiated with an RCP laser at a wavelength of 1550 nm, where 2D EDS mapping was performed at an accelerating voltage of 10 kV. **Figure S6b** presents the 2D mapping obtained from the Ti Kα line (4.508 keV), in which the S-shaped nanostructure is clearly visible. **Figure S6c** shows the 2D mapping obtained from the Zn Lα line (1.012 keV). These elemental mappings confirm that the products formed on the S-shaped nanostructures under laser irradiation contain zinc (Zn).

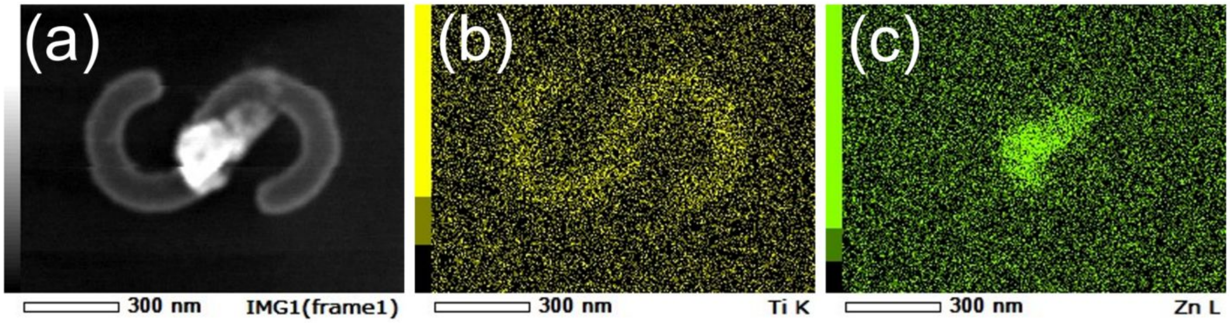

Figure S6. 2D EDS mapping of the S-shaped nanostructure irradiated with an RCP laser at a wavelength of 1550 nm. (a) SEM image of the observed area during the EDS measurement. (b) 2D mapping of Ti. (c) 2D mapping of Zn.

S6. Steady-state temperature distributions of S-shaped nanostructures with total lengths of 400 nm and 1100 nm.

To examine the effect of size on chiral plasmonic temperature switching, we calculated the steady-state temperature distributions of S-shaped nanostructures with different sizes. The calculation method and the light intensity used were the same as those in **Figure 2** of the main article. **Figures S7a** and **S7c** show the temperature distributions of S-shaped nanostructures with a total length of 400 nm and a line width of 50 nm under RCP and LCP illumination. Compared with the 770 nm-long S-shaped structure shown in **Figure 2** of the main article, the contrast of chiral temperature switching is more pronounced. However, due to limited opportunities for electron-beam lithography, fabricating such small structures was challenging from a resolution standpoint. **Figures S7b** and **S7d** show the temperature distributions of larger S-shaped nanostructures with a total length of 1100 nm and a line width of 100 nm under RCP and LCP illumination. In these somewhat larger structures, the contribution of localized plasmons to light absorption is reduced, resulting in smaller temperature contrasts. For these reasons, we conducted the demonstration experiments using an intermediate size, the 770 nm-long S-shaped nanostructure.

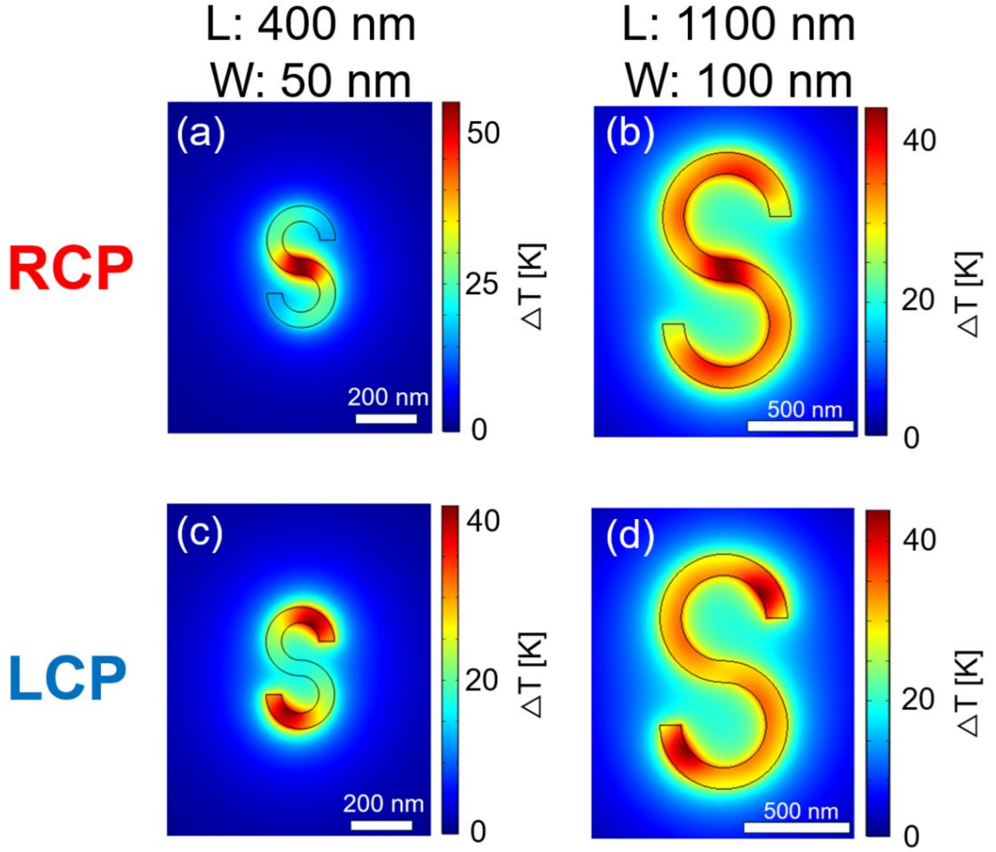

Figure S7. (a, c) Steady-state temperature distributions of TiN S-shaped nanostructures with a total length of 400 nm and a line width of 50 nm under RCP and LCP illumination. (b, d) Steady-state temperature distributions of TiN S-shaped nanostructures with a total length of 1100 nm and a line width of 100 nm under RCP and LCP illumination. In all calculations, the irradiance was the same as in **Figure 2** of the main article, 1.0×10^{10} W m^{-2}.
